# Different MRI structural processing methods do not impact functional connectivity computation

**DOI:** 10.1038/s41598-023-34645-3

**Published:** 2023-05-26

**Authors:** Lu Zhang, Lorenzo Pini, Maurizio Corbetta

**Affiliations:** 1 Padova Neuroscience Center, University of Padova, 35131 Padua, Italy; 2 Venetian Institute of Molecular Medicine (VIMM), 35129 Padua, Italy; 3 Clinica Neurologica, Department of Neuroscience, University of Padova, 35131 Padua, Italy

**Keywords:** Neuroscience, Cognitive neuroscience, Computational neuroscience

## Abstract

Resting-state functional magnetic resonance imaging (rs-fMRI) has become an increasingly popular technique. This technique can assess several features of brain connectivity, such as inter-regional temporal correlation (functional connectivity), from which graph measures of network organization can be derived. However, these measures are prone to a certain degree of variability depending on the analytical steps during preprocessing. Many studies have investigated the effect of different preprocessing steps on functional connectivity measures; however, no study investigated whether different structural reconstructions lead to different functional connectivity metrics. Here, we evaluated the impact of different structural segmentation strategies on functional connectivity outcomes. To this aim, we compared different metrics computed after two different registration strategies. The first strategy used structural information from the 3D T1-weighted image (unimodal), while the second strategy implemented a multimodal approach, where an additional registration step used the information from the T2-weighted image. The impact of these different approaches was evaluated on a sample of 58 healthy adults. As expected, different approaches led to significant differences in structural measures (i.e., cortical thickness, volume, and gyrification index), with the maximum impact on the insula cortex. However, these differences were only slightly translated to functional metrics. We reported no differences in graph measures and seed-based functional connectivity maps, but slight differences in the insula when we compared the mean functional strength for each parcel. Overall, these results suggested that functional metrics are only slightly different when using a unimodal compared to a multimodal approach, while the structural output can be significantly affected.

## Introduction

Resting-state functional magnetic resonance imaging (rs-fMRI) is a popular imaging approach aimed at studying the intrinsic activity of the brain during rest. Brain regions showing temporal correlation of the blood oxygen level dependent (BOLD) signal are considered to be functionally connected into intrinsic resting state networks^1–6^. Rs-fMRI functional connectivity (FC) is emerging as an important feature for both basic neuroscience and clinical researchers, shedding new light on the functional organization of the human brain. Moreover, in the last two decades, a large amount of literature indicates that FC contributes to cognitive, sensory, and social functions. A piece of evidence comes from clinical studies, showing that network breakdowns are related to cognitive deficits^7–15^.

However, the application of rs-fMRI is still limited by several factors, including preprocessing. To date, there are no gold standard procedures for fMRI processing, limiting the possible application of this tool to the clinical arena. Moreover, the field suffers from a reproducibility crisis. Recently, a large collaborative study reported low reproducibility of results among 70 independent international teams working on the same fMRI dataset. Although this result was obtained based on task-state fMRI, not rs-fMRI, this^16^ and other studies showed that the results strongly depend on the decisions made in terms of preprocessing^17–21^. This problem is also evident in other methods such as diffusion magnetic resonance imaging and arterial spin labeling that use similar acquisition protocols, e.g., echo-planar imaging^22–24^. On the contrary, structural imaging assessed with T1-weighted sequences (T1w) shows greater reproducibility between sites and vendors^25^. However, results from structural imaging are not completely independent of preprocessing. Different parameters can impact the results. For instance, the FreeSurfer pipeline, one of the most popular toolboxes for the analysis of brain surface morphology^26^, shows some limitations, as observed by Gronenschild et al. reporting a significant impact of these variables on structural MRI (sMRI) computation. That is, using different software versions (v4.3.1, v4.5.0, and v5.0.0), workstations (Macintosh and Hewlett-Packard), and operating systems (OSX 10.5 and OSX 10.6) they reported an average significant difference of around 9% and 3% for volume and cortical thickness, respectively^27^. This result must be considered in the context of fMRI analysis as sMRI scans are used for regressing non-neural signals (i.e., used to identify white matter (WM) and ventricle voxels) and for registering data into a specific geometrical space (e.g., T1w-native or standard space). FC measures, such as graph theoretical measures^28^, may differ based on the different preprocessing steps selected.

In recent years, several attempts have been made to improve sMRI segmentation. While surface reconstruction is mostly computed using a single T1w scan, it is possible to exploit the information from sMRI data employing longer repetition time (TR) and echo time (TE), such as T2-weighted (T2w) and fluid-attenuated inversion recovery (FLAIR) images. T1w data enables the segmentation of gray matter (GM) and WM, while T2w enhances the signal of the water. Previous studies used T1w and T2w images in combination to refine the placement of the pial surface, to exclude nonbrain regions from T1w images, and to correct the bias field of the images^29,30^. Moreover, Glasser et al.^31^ improved surface reconstructions and myelin maps based on the T1w/T2w ratio. Similarly, FLAIR sequences can improve anatomical information. This statement has been demonstrated by Lindroth et al. reporting significant improvement in surface reconstruction by combining T1w and FLAIR through the FreeSurfer pipeline^32^.

However, it is unclear whether the combination of T1w and T2w signals can affect FC metrics due to differences in the preprocessing steps. This influence can occur both during the creation of the GM and WM masks used for computing non-neural signal regression or during imaging co-registration steps. Based on these premises, we aimed at investigating whether different structural reconstruction strategies would impact FC connectivity outcomes within the context of surface FC. Specifically, we evaluated whether a multimodal reconstruction approach (combining T1w and T2w images; referred to as multimodal pipeline—MP) would lead to different FC outcomes compared to a unimodal approach (using only information from T1w image; referred to as unimodal pipeline—UP).

## Results

**Figure 1:**
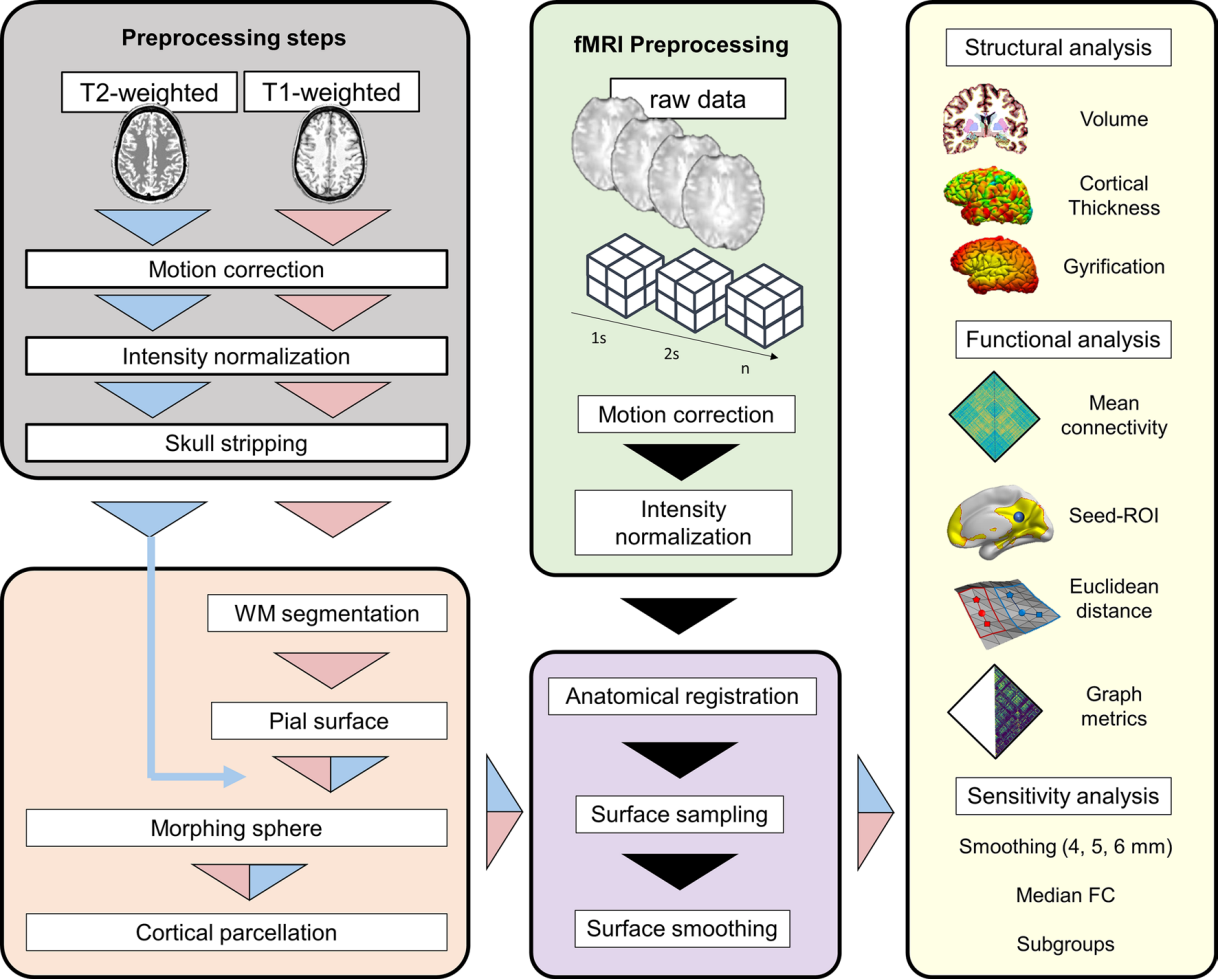
Workflow of the analysis. Each participant underwent resting-state functional, T1-weighted (T1w), and T2-weighted (T2w) MRI scans. Surface reconstruction was performed through a unimodal pipeline (UP; using T1w signal) or a multimodal pipeline (MP; combining T1w and T2w signals). For the UP workflow (pink triangle), 3D T1w structural images were processed using the recon-all processing stream, which performed all reconstruction steps, including motion correction, intensity normalization, skull-stripping, white matter (WM) segmentation, spherical morph, and parcellation. MP workflow (blue and pink triangle) included the T2w images (blue triangle) in the Freesurfer workflow, recomputing spherical morph and cortical parcellation to adjust the pial surfaces. The preprocessing steps for resting-state data included head movement correction, intensity normalization, anatomical registration, and smoothing. For the anatomical registration step, the rs-fMRI preprocessing pipeline was run independently for the UP and the MP. Structural (volume, cortical thickness, and gyrification index) and functional (mean FC, seed-ROI maps, spatial topology, and graph analysis) outcomes were compared between the two different pipelines.

The whole workflow is reported in Fig. 1, describing the analytical procedure and the analyses performed in the study.

### Structural outcomes

**Figure 2:**
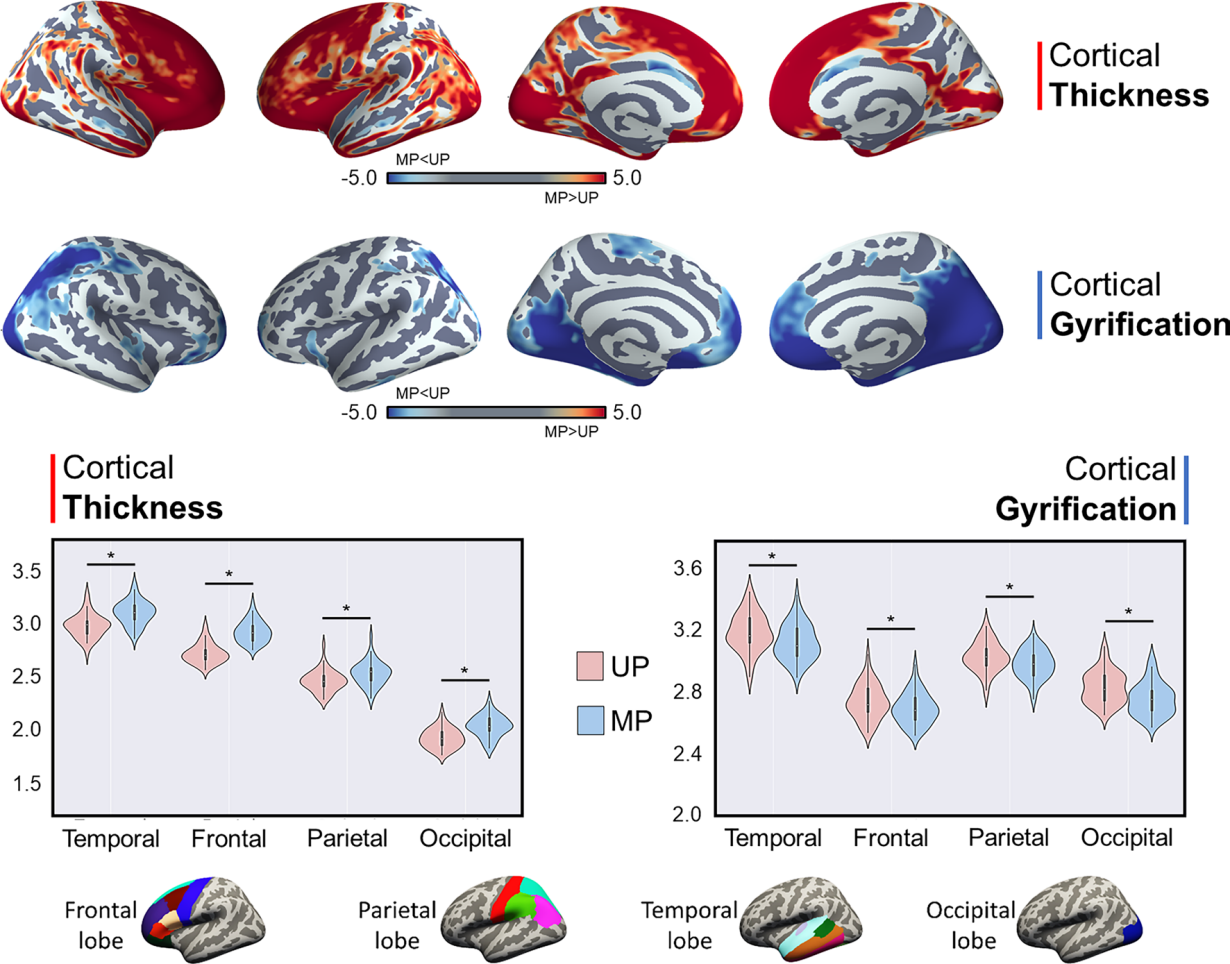
Structural differences between unimodal and multimodal pipelines. Top panel: difference in cortical thickness at the vertex level, stronger within the bilateral insula (first row); differences in the gyrification index at vertex level (second row). Middle panel: significant differences in cortical thickness (left) and gyrification index (right) within frontal, parietal, temporal, and occipital cortices were found between the two processing modalities. Significantly thicker cortical thickness was reported for the multimodal pipeline, compared to the unimodal pipelines. Significantly higher gyrification index was reported for the unimodal pipeline, compared to the multimodal pipelines. Bottom panel: subdivision of the Desikan–Killiany atlas parcels for the four lobes.

In line with previous studies, we reported a significant difference in cortical thickness between the two pipelines. This difference, widespread to the cortex, was stronger for the insula (see Fig. 2). MP led to a significantly widespread thicker surface, except for the bilateral caudal anterior cingulate cortex (ACC), left superior temporal gyrus, and right superior parietal lobule (*p*_*Bonferroni*_ > 0.0014) (Supplementary Table S1). Similarly, a higher cortical volume was reported for the MP compared to UP, except for left ACC, left posterior cingulate cortex, right cuneus, right lingual gyrus, and bilateral superior parietal lobule (*p*_*Bonferroni*_ > 0.0014) (Supplementary Table S2). For subcortical regions, results were mixed, with MP showing higher volumes for the bilateral amygdala, and the right accumbens, while UP led to higher bilateral thalamus volumes. These results were confirmed in the subgroup of individuals acquired with the same MRI protocol; the z-maps expressing cortical thickness difference between UP and MP for the whole cohort and the subgroup (n = 44) showed a high and significant correlation (*r* = 0.83; *p* < 0.001) suggesting that the difference reported is ubiquitous to different MRI parameters. Additionally, we reported a significant difference in the gyrification index between the MP and UP. MP led to a significantly lower gyrification index widespread across the cortex, while few regions did not survive multiple comparison significance (bilateral caudal middle frontal gyrus, bilateral transverse temporal gyrus, left ACC, left pars opercularis, left pars orbitalis, left precentral gyrus, left posterior cingulate cortex, right banks of the superior temporal sulcus and right middle temporal gyrus) (Supplementary Table S3). The vertex-wise analysis confirmed this pattern (see Fig. 2). A post-hoc analysis was performed to assess whether these results may be driven by a pipeline-dependent vertices-to-parcels assignment. The Jaccard coefficient for the left insula—which exhibited the greatest difference in structural outcome between UP and MP—was found to be high (mean J = 0.90 ± 0.01), indicating a comparable spatial pattern in vertex assignment between the two pipelines. The very slight difference in vertex assignment alone is unlikely to account for the reported overall differences.

### Functional connectivity outcomes

**Figure 3:**
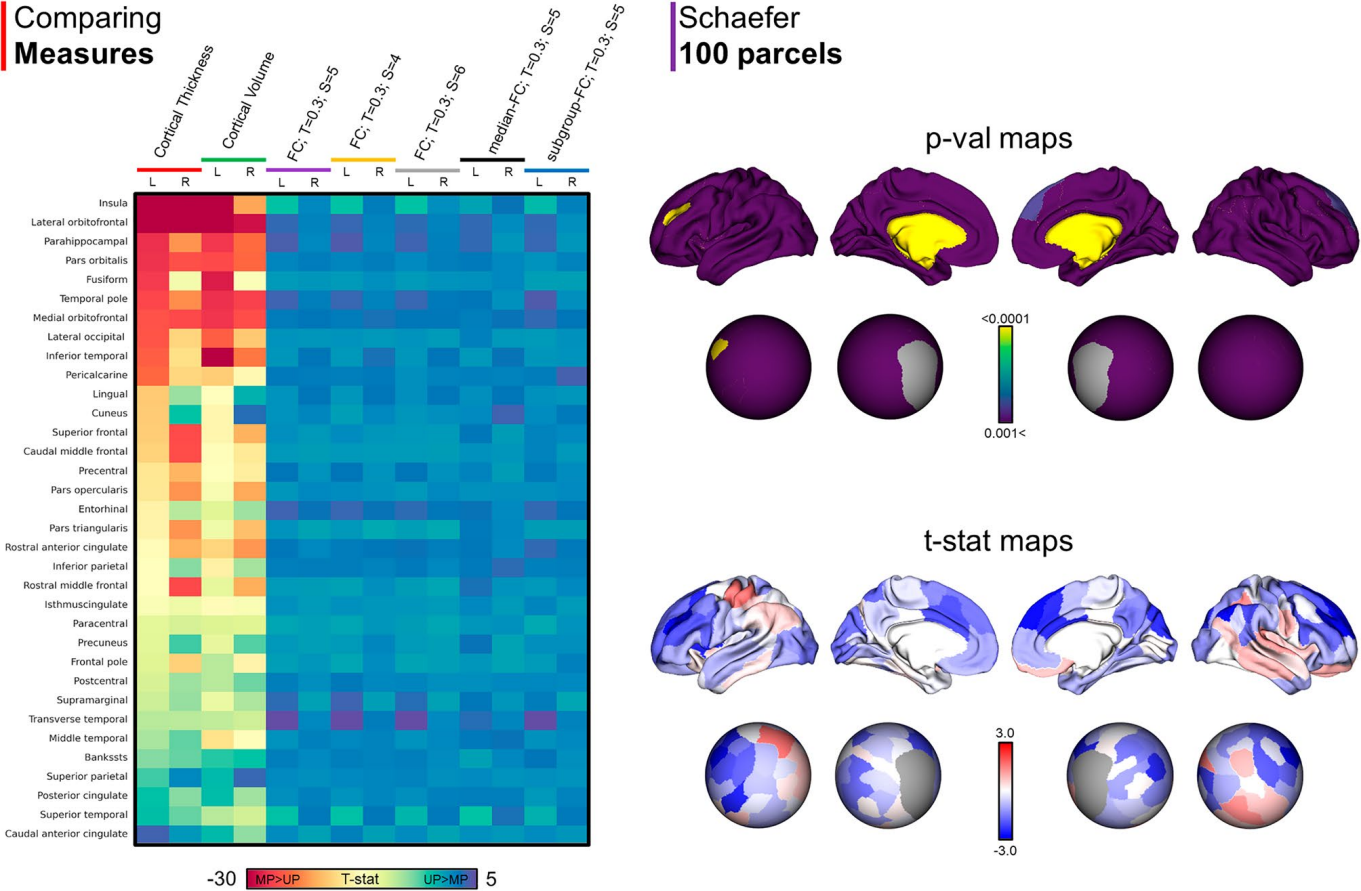
Functional connectivity differences between unimodal and multimodal streams. Left panel: differences in cortical thickness, volume, mean functional connectivity (FC) with different smoothing parameters (Gaussian smoothing kernels: 5 mm, 4 mm, and 6 mm), median FC, and mean FC computed using a subcohort of participants acquired with the same MRI protocol. Regions were reordered according to *t*-values from the paired sample *t*-test cortical thickness analysis (left hemisphere). The left insula was the parcel with the highest t value (absolute value); the left anterior cingulate (ACC) was the parcel with the lowest t value (absolute value). Right panel: differences in mean FC between unimodal and multimodal streams projected to the Schaefer 100-parcel atlas. A significant difference was reported only for the left prefrontal cortex. *T:* threshold, *S:* Gaussian smoothing kernels.

Contrary to the widespread differences for cortical thickness and volumes, mean FC values computed with MP and UP were not significantly different except for the left insula (*t* = − 3.599, *p* = 0.0007), left superior temporal gyrus (*t* = − 3.503, *p* = 0.0009) and the left transverse temporal region (*t* = 3.504, *p* = 0.0009). As for the structural outcomes, the analysis limited in the subgroup of individuals acquired with the same MRI protocol (n = 44) confirmed these results, showing a significant difference between UP and MP limited to the left insula (*t* = − 3.113, *p* = 0.003) and the left transverse temporal region (*t* = 2.929, *p* = 0.006). Similar results were reported computing FC as the median of values in the whole cohort, which showed a significant difference only in the left superior temporal gyrus (*t* = − 3.646, *p* = 0.0006). These results were atlas-independent as using a different brain parcellation scheme (Schaefer 100 parcel atlas) FC mean values were similar between UP and MP across the whole cortex, except for a single parcel (left prefrontal cortex; *t* = − 4.604, *p* < 0.0001; Fig. 3) surviving after multiple comparison correction. Finally, we tested the effect of different smoothing parameters on the rs-fMRI to assess for potential confounding factors. We reported similar results in the left insula (4-mm FWHM Gaussian kernel: *t* = − 3.635, *p* = 0.0006; 6-mm FWHM Gaussian kernel: *t* = − 3.645, *p* = 0.0006), in the left transverse temporal region (4-mm FWHM Gaussian kernel: *t* = 3.608, *p* = 0.0007; 6-mm FWHM Gaussian kernel: *t* = 3.374, *p* = 0.001) and in the left superior temporal gyrus (4-mm FWHM Gaussian kernel: *t* = − 3.455, *p* = 0.001; 6-mm FWHM Gaussian kernel: *t* = − 2.884, *p* = 0.006), although the difference in the left superior temporal gyrus was not significant after Bonferroni correction (*p* < 0.0014) with a Gaussian kernel equaled to 6 mm (Fig. 3). These results were partially echoed by the FC seed-vertex region of interest (ROI) group analysis for the left and right five networks, showing no significant differences between MP and UP at the vertex level.

### Region of interest connectivity results

**Figure 4:**
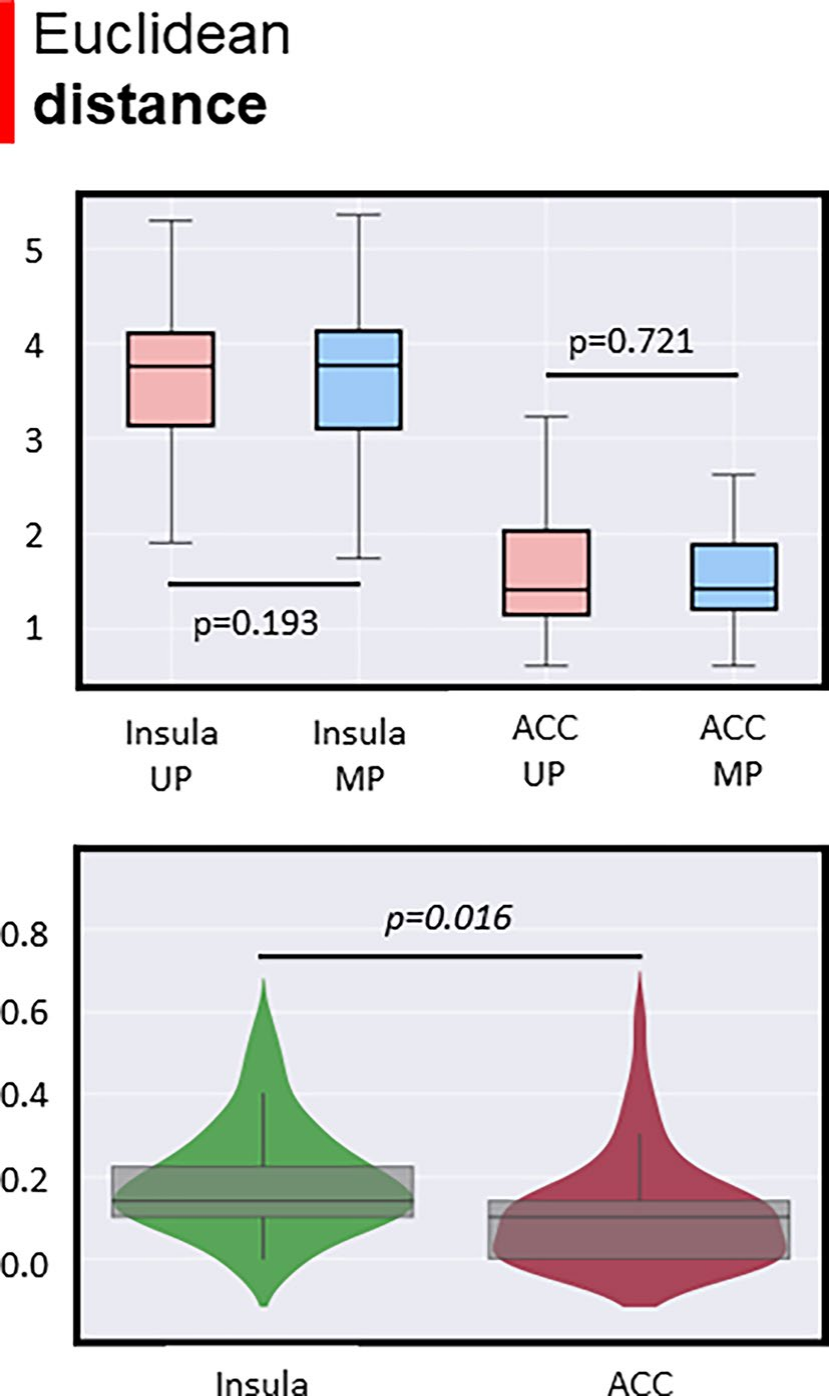
Spatial topological differences between unimodal and multimodal streams. The left insula and left anterior cingulate cortex (ACC) were selected as regions of interest (ROIs) and the Euclidean distance in the 3D anatomical space between ROI’s center of gravity and peak FC voxels was computed. Top panel: Euclidean distance did not report significant differences between unimodal and multimodal streams. Bottom panel: Significant differences were observed between the delta shift (Euclidean distance) of the left insula and the left ACC.

According to the cortical thickness results (see Fig. 3), parcels with the highest and lowest *t value,* left insula and left ACC respectively, were selected as ROIs. The Euclidean distance for each ROI between MP and UP did not report significant differences (left insula: *p* = 0.193; left ACC: *p* = 0.721) (Fig. 4) suggesting that FC map peak was overall located in the same spatial voxels. By contrast, when we compared the delta Euclidean distance between the insula and ACC, we found a significant difference (*p* = 0.0016) (Fig. 4). This result suggested that a slight shift of the insula FC peak was bigger than the difference of the ACC, suggesting a slight translational effect of the structural findings in FC.

**Figure 5:**
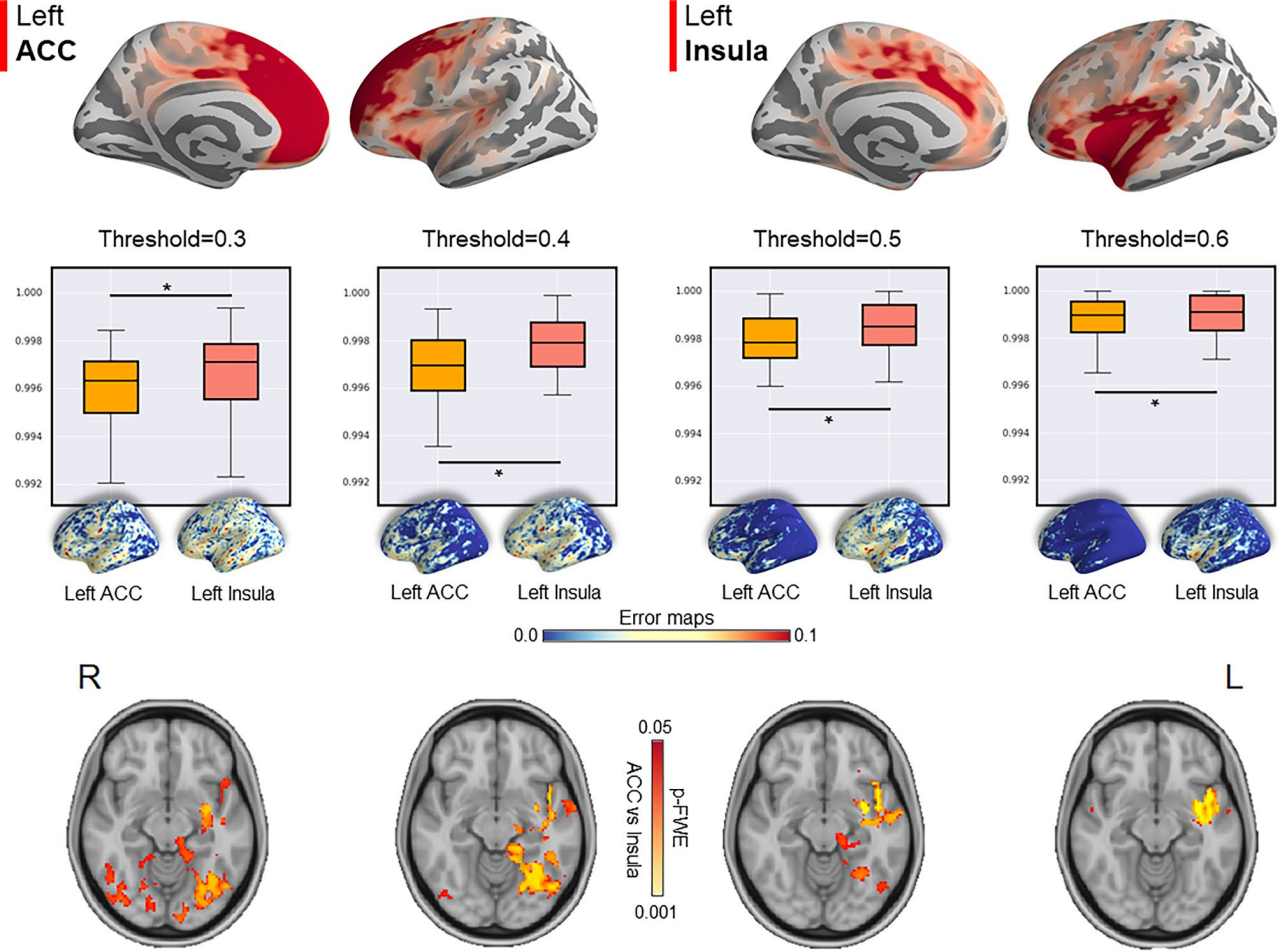
Difference in FC topography between unimodal and multimodal streams. Top panel: mean seed-based connectivity maps for the left insula (left) and left anterior cingulate cortex (ACC) (right). Middle panel: Significant differences between the insula and ACC error maps (computed as the difference between UP and MP). Higher values mean higher errors. Bottom panel: Significant differences between the insula and ACC error maps were found at the voxel level.

When we compared error maps (MP vs UP) between ACC and insula, we found a similar spatial distribution. Specifically, error maps showed overall low error values, suggesting that FC maps computed with MP and UP were similar. However, slight differences were found, specifically for temporal and frontal regions (see Fig. 5). When we computed an overall error value (mean error values across voxels) we found a significant difference between the left insula and left ACC, suggesting that the parcel with the highest structural difference showed also the lowest similarity between the two pipelines at each threshold considered (FC threshold 0.3: *p* = 0.0058; FC threshold 0.4: *p* = 0.0017; FC threshold 0.5: *p* = 0.0011; FC threshold 0.6: *p* = 0.0028) (Fig. 5). When this difference was investigated at the voxel-wise level, we found a significant difference with a maximal expression in the left insula (Fig. 5).

### Graph connectivity patterns

**Figure 6:**
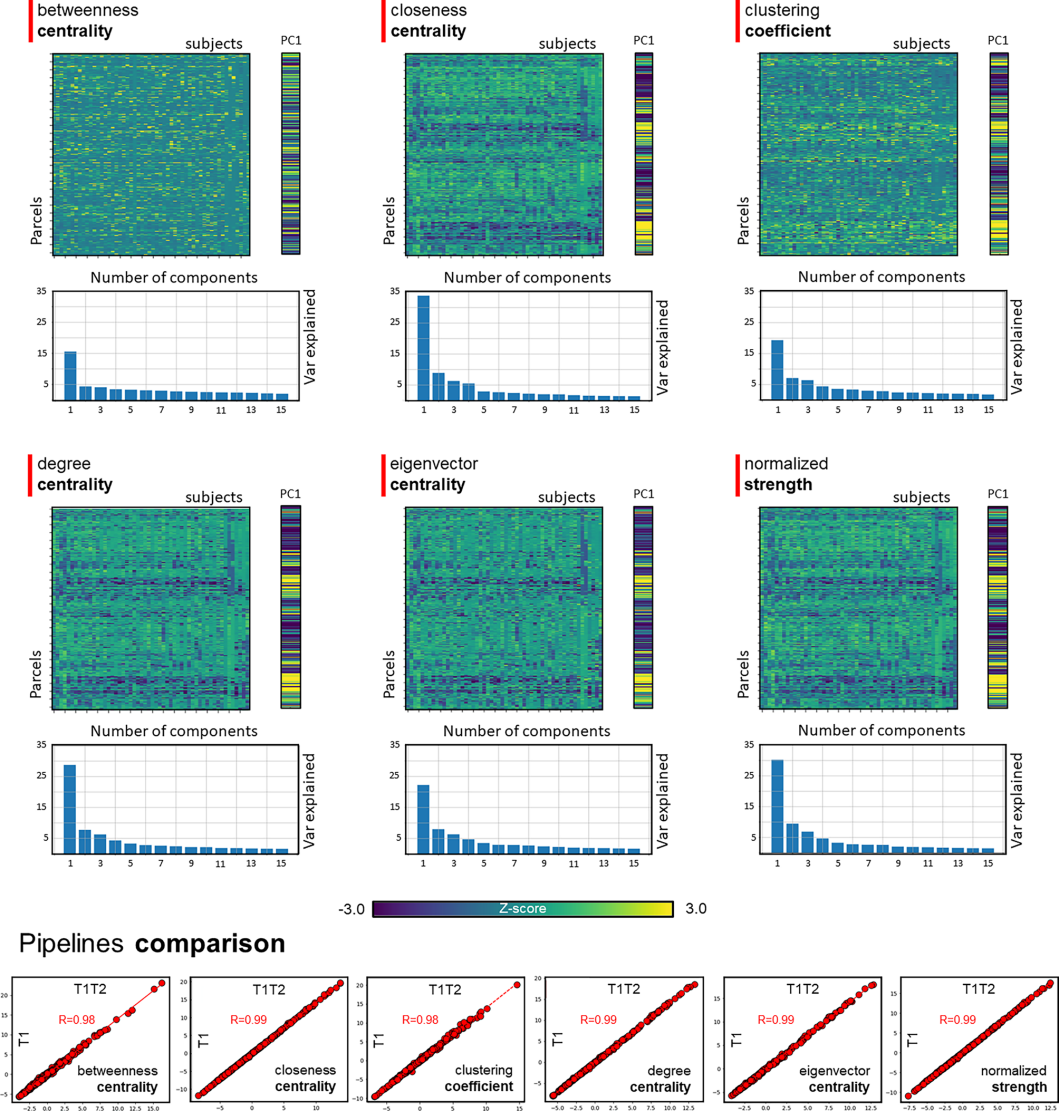
Graph theory metrics differences between unimodal and multimodal streams. Betweenness centrality, closeness centrality, clustering coefficient, degree centrality, eigenvector centrality, and normalized strength were computed for each parcel and each subject. Outcomes were organized into a j × k matrix (j = the number of parcels; k = the number of participants) and fed to a principal component analysis. The first component (PC1) was selected and compared between unimodal and multimodal streams. PC1 from UP and MP showed the same structure for the matrix (*r* > 0.98).

We compared the first principal component of each graph metric for the rs-fMRI processed data from the UP and MP. The first principal componence from UP was highly correlated with the first principal componence from MP (betweenness centrality *r* = 0.98, closeness centrality *r* = 0.99, clustering coefficient *r* = 0.98, degree centrality *r* = 0.99, eigenvector centrality *r* = 0.99, and normalized strength *r* = 0.99) (Fig. 6), in line with mean FC results.

## Discussion

In the present study, we evaluated the impact of a preprocessing imaging pipeline combining T1w and T2w signals compared to a unimodal approach considering only the signal from the T1w image. In line with the previous literature, we reported a significant difference in structural metrics, even in a relatively small sample size of 58 subjects. The multimodal pipeline led to a significantly thicker cortical thickness, higher volume values, and lower gyrification index compared to the unimodal processing. These differences were widespread across the cortex. Note, the negative relationship between higher cortical thickness and lower gyrification for the MP pipeline is in line with previous literature reporting a negative correlation between these two measures^33,34^. However, these differences were only slightly translated in the FC metrics, suggesting a limited impact on FC computation metrics, while graph metrics were not affected.

The effect of combining T1w and T2w/FLAIR has previously been assessed^35^. A significant advantage of this multimodal pipeline was demonstrated for the computation of structural outcomes linked with cortical segmentation^32,35–37^. The additional value of the structural MP was reported for both healthy individuals, and patients and for the identification of age-related cortical atrophy^32^. Our results are perfectly in line with these studies. We observed a widespread and significant difference between MP and UP approaches. This is not unexpected, since the simultaneous use of T2w and T1w images can solve the misclassification of vessels, dura, and gray matter through multiple signal sources^35^.

However, these differences were not reported in FC computation. We reported a limited impact on connectivity metrics, ranging from static FC, similarity values, and graph analysis. Notably, using the insula as a seed ROI showed overlapping topological results, suggesting that the FC computation might be, at least partially, independent of the structural processing pipeline. These results did not suggest that different processing parameters for fMRI data did not influence subsequent FC analysis, since UP and MP were equivalent in terms of preprocessing strategy, except for the structural source of the signal. Why did a huge difference in structural segmentation lead to a slight difference in FC? fMRI preprocessing involves several steps, such as motion correction, spatial normalization, and spatial smoothing, involving interpolation procedures. These transformations might reduce the potential differences introduced by the inclusion of different structural signals. The Gaussian smoothing kernel is the most commonly implemented procedure in fMRI^38^. This step is an important operation, although its implementation is controversial. Some amount of smoothing is applied to ‘blur’ images, increasing the signal-to-noise ratio and removing residual noise after spatial registration. However, when different smoothing parameters were applied to the rs-fMRI data we observed comparable results, suggesting a limited impact of smoothing on the data, although spatial smoothness has been identified as one of the most common analytical factors related to different fMRI results^16^.

However, we found a slight but still significant effect within the insula, orbitofrontal cortex, and temporal cortex, the regions showing the highest differences in terms of structural outcomes (both volume and thickness). These regions are the most prone to error during automatic segmentation^39,40^. Although our analysis cannot assume that cortical thickness and volume reconstruction through combining T1w and T2w signals is better than unimodal pipeline, these results should be interpreted in light of previous findings, suggesting that clinical studies might benefit from a combination of multiple sources of MRI structural signal. This might be relevant for clinical neuroimaging studies assessing structural features in brain disorders, for which MRI computation can be affected by atrophy. Notably, we reported that FC similarity maps within the insula showed higher error scores, compared to a parcel with the lowest difference in terms of structural outcome. The insula is known to play a key role in neurodegenerative disease^13,41–46^ and psychiatric disorders^47–51^. Moreover, this brain structure is linked with the connectivity of the SN, a network involved in social, emotional, and attentional cognition. Recent evidence reported that dysregulation of SN occurs in many brain conditions, including autism spectrum disorder^52^, psychosis^53^, and FTD^54^, all conditions sharing deficits in social cognition, such as the ability to infer other people’s mental states, thoughts, and feelings and referred to as ‘theory of mind’^55,56^. Within this framework, our results would be useful in guiding clinical neuroimaging studies focused on this network/structure, for which the pipeline of analysis should be selected with caution, considering the trade-off between improving the MRI signal and the time to acquire additional MRI structural scans. Overall, our results suggested that researchers should be careful with FC maps obtained from different pipelines, although we do not believe that there is an optimal pipeline in all studies.

Results from the present study should be interpreted in light of some limitations. The sample size was relatively small compared to the large dataset available in the literature. However, we investigated whether differences would affect a cohort of healthy participants usually reported in fMRI studies (numerosity around 40/50 participants). Additionally, our MRI data were obtained from different MRI scanners/parameters, although the analysis performed on a subgroup of individuals acquired with the same MRI protocol showed comparable results (Supplementary Fig. S1). Moreover, we did not consider whether other preprocessing parameters would affect the UP and MP approach, except for the smoothing amount parameter. Further studies should investigate whether different preprocessing parameters would lead to widespread differences also for FC outcomes using these different structural strategies. Finally, the estimate of FC can be influenced by physiological noise, such as cardiac and respiratory fluctuations. The impact of physiological noise removal and shorter TR on different pipelines should be evaluated by further studies.

In conclusion, this study extends the results of previous studies, suggesting that structural reconstruction through a UP or MP approach, contrary to other fMRI preprocessing steps, such as temporal filtering, and motion correction^20,21^, has a very limited impact on functional metric computation. On the contrary, these two approaches led to significant results in terms of structural outcomes. However, the slight difference in functional output for the insula would suggest caution when the focus of the study is on the investigation of the brain scaffold related to this brain structure and for clinical studies focused on pathology related to insula vulnerability.

## Methods

### Participants

Fifty-eight healthy adults (33 females, mean age 27.4) were retrospectively included in this analysis. All participants were right-handed, had normal or corrected to normal vision, and had no history of neurological or psychological diseases. These participants were pooled from different cohorts^57–59^. All participants signed an informed consent form before the study and were compensated for their participation. These studies were approved by the Institutional Review Board (IRB) of Washington University in St. Louis School of Medicine. All methods used in the current study were performed in accordance with the relevant guidelines and regulations of the ethical review board.

### MRI acquisition

MRI data were acquired using a Siemens (Erlingen, Germany) 3-T Prisma Fit MR scanner with a 32-channel RF head coil. Structural images were acquired using 3D T1-weighted Magnetization Prepared Rapid Gradient Echo (MPRAGE) (1 mm isotropic voxel; TE = 2.36 ms, TR = 1700 ms, Inversion Time (TI) = 1000 ms, flip angle (FA) = 8°) and 3D T2-weighted fast spin echo sequences (1 mm isotropic voxel; TR/TE = 3200/564 ms, FA = 120°). Resting-state fMRI scans were collected using a gradient echo-planar sequence sensitive to BOLD contrast (2.4 mm isotropic voxels, TE = 32.4 ms, TR = 1 s, FA = 63°, 48 slices, and multiband factor 4). Seven participants underwent MRI examination with different parameters (3D T1-MPRAGE: 1 mm isotropic voxel, TR/TE = 2400/2.22 ms, TI = 1000 ms, FA = 8°; 3D T2-weighted: 0.8 mm isotropic voxel, TR/TE = 3200/563 ms, FA = 120°; resting-state fMRI: 3 mm isotropic voxel, TE = 25.8 ms, TR = 1 s, FA = 58°, 56 slices and a multiband factor of 3). Seven participants were acquired using a Siemens 3-T Tim Trio MR system with a 16 channel RF head coil (3D T1-MPRAGE: 1 mm isotropic voxel, TR/TE = 1950/2.26 ms, TI = 900 ms, FA = 9°, and 176 sagittal slices; 3D T2-weighted: 1 mm isotropic voxel, TR/TE = 2500/442 ms, FA = 120°; resting-state fMRI: 4 mm isotropic voxel: TR = 2 s, TE = 27 ms, FA = 90°, 32 interleaved slices.

All participants underwent three resting-state fMRI runs, each lasting 5 min (300 or 150 TRs), for a total of 15 min. During resting state scans, participants were asked to maintain fixation on a cross that was displayed in the center of the screen.

### Structural and functional data processing

Surface-based cortical measurements were obtained using FreeSurfer v7.1.1 (https://surfer.nmr.mgh.harvard.edu/). First, 3D T1-weighted structural images were processed using a recon-all processing stream, which included motion correction, skull-stripping, registration, cortical and subcortical segmentation, smoothing, spherical morph, and parcellation mapping (see Fig. 1 for the workflow of the structural preprocessing). Quality checks were visually performed to ensure the accuracy of segmentation and surface reconstruction. Cortical parcellation statistics were extracted using the Desikan-Killiany Atlas (DKT), which contains 68 regions, 34 regions in each hemisphere.

Resting-state data were processed through the FSFAST utility (https://surfer.nmr.mgh.harvard.edu/fswiki/FsFast) implemented in Freesurfer. The preprocessing steps included: the removal of the first 4 scans for each resting state run to allow for magnetic field stabilization; template creation; brain mask extraction; rs-fMRI registration to anatomical images; head movement correction; slice-timing correction; spatial normalization; resampling time series to the left and right surfaces; spatial smoothing at 5-mm full width at half maximum (FWHM) Gaussian kernel. Finally, for each participant, the different resting state runs were concatenated for the FC analysis.

Structural (cortical thickness, gyrification, and parcel volumes) and functional (mean FC, seed-ROI maps, spatial topology, and graph analysis) outcomes were compared between the two pipelines: the UP considered only the signals from T1w images for surface and volumetric brain computation; the MP included the signal from the T2w image in the Freesurfer workflow, recomputing spherical morph and cortical parcellation to adjust the pial surfaces previously computed using only the T1w data. The rs-fMRI preprocessing pipeline was run independently for the UP (i.e., using the surface reconstruction from T1w-image) and the MP (i.e., using the surface computed combining T1w and T2w information (see Fig. 1).

### Volumes, cortical thickness, and gyrification index

Volumetric and cortical thickness metrics were compared between MP and UP. While the former refers to the number of voxels/vertices within a specific parcel, the latter is a measure of the width of the GM, computed as the closest distance from the gray-white surface to the gray-cerebrospinal fluid boundary (pial surface) at each vertex^26,40,60^. A paired sample *t*-test was performed for cortical thickness and volumes, corrected for Bonferroni comparison (alpha significance of 0.05/n, where n represents the 34 parcels for each hemisphere). Similarly, we compared subcortical volumes (Bonferroni correction threshold 0.05/n; n = 7 subcortical regions). This analysis was repeated at the vertex level (surface-based group analysis; multiple comparison correction—Monte Carlo simulation—with vertex-wise threshold *p* < 0.0001, and cluster-wise *p*-value < 0.05, 5000 iterations). Finally, we computed the gyrification index^61^, a structural metric that is defined as the ratio of the total folded cortical surface over the amount of cortex on the outer visible cortex. Differences in the gyrification index for each DKT region were compared using paired sample *t*-tests Bonferroni corrected (0.05/34). The same vertex analysis applied to cortical thickness was performed for the gyrification outcomes.

### Functional strength of the whole brain

All the rs-fMRI analysis was performed at the surface level, not involving subcortical areas. For each parcel (from the DKT atlas) we extracted the corresponding timeseries (averaged timeseries of each vertex belonging to that parcel). A parcel-wise connectivity matrix was then computed (Pearson’s correlations). This procedure led to a symmetric matrix (68 parcels × 68 parcels) for each participant. Diagonal values were removed as they represented the correlations between the parcels and themselves (ones). We also removed values with an absolute value lower than 0.3, unlikely to represent connections. Finally, each column of this cleaned matrix was averaged, representing the mean FC strength of that parcel. The procedure was repeated for the rs-fMRI data obtained using the MP and the UP. We then investigated whether mean FC values were independent of the preprocessing stream by comparing mean MP and UP FC outcomes through a paired-sample *t*-test (Bonferroni corrected for multiple comparisons—0.05/34). We performed an additional analysis considering the median instead of the mean since the former measure is less prone to potential outliers. Finally, we assessed whether different smoothing values would impact the FC outcomes. The full preprocessing was repeated considering additional smoothing kernels of 4 mm, and 6 mm (in addition to 5 mm, considered the standard parameter in this analysis). Mean FC connectivity was then computed on these preprocessed images.

We selected five DKT parcels from the left and right hemisphere as ROIs, representing five main resting-state networks: (i) insula—salience network (SN); (ii) parahippocampus—limbic network (LIM); (iii) pericalcarine gyrus—visual network (VIS), (iv) precentral gyrus—somatomotor network (SMN); (v) isthmus cingulate cortex—default mode network (DMN). The averaged timeseries from each ROI were extracted to compute Pearson’s correlations between these ROIs and surface vertices within the same hemisphere. A paired surface-based group analysis was performed to compare FC strengths for the 5 networks between MP and UP (multiple comparisons—Monte Carlo simulation: vertex-wise threshold *p* < 0.0001, cluster-wise *p* < 0.05, cluster size > 50 mm^2^, 5000 iterations).

### Functional region of interest analysis

We performed two different analyses based on the two extremes of the axis expressing differences in terms of cortical thickness. The first analysis was limited to the left hemisphere and at the voxel level (registering vertex maps to voxels using the inverse of the transformation matrix to register fMRI data to the surface). We selected the parcels showing the highest and the lowest cortical thickness difference between the MP and UP streams. As in the network analysis, these parcels were used as ROI to compute parcel-voxels correlations within the left hemisphere. The corresponding FC maps were then compared between MP and UP. For each participant and pipeline stream, we estimated the single voxel showing the FC peak. We then computed the Euclidean distance between the peak 3D location and the center of gravity of the ROI. This procedure was repeated for data computed with both MP and UP and compared using a paired sample *t*-test. Significant results would indicate a spatial shift of the FC peak between the two processing streamlines. In the second analysis, the FC maps (from both hemispheres) were compared directly between MP and UP as follows: (i) for each ROI the corresponding FC maps were binarized by a set threshold (from 0.3 to 0.6, the step of 0.1); (ii) error maps between binarized FC maps computed with MP and UP were calculated. The mean values of these error maps were compared using a paired sample *t*-test. Finally, we compared spatially the differences between error maps (smoothed with a sigma of 2.12 mm) through a non-parametric procedure (threshold-free enhanced cluster; TFCE with a *p*-Family-Wise Error (FWE) correction < 0.05, *n* = 5000 permutations).

### Graph analysis

We investigated whether whole-brain graph metrics would exhibit differences between MP and UP. We used the 333 parcels specified by Gordon et al.^62^ representing the node of a graph, while FC between each pair of parcels was defined as an edge. For each participant and processing stream, we calculated the FC matrix representing Pearson’s timeseries correlations between each pair of parcels. The correlation values were Fisher's transformed. The matrix was thresholded (r = 0.3) and binarized it. Six graph properties, betweenness centrality, closeness centrality, degree centrality, eigenvector centrality, clustering coefficient, and normalized strength were computed^63^. For each measure, graph values at the parcel level of each participant were concatenated in a j × k matrix (j = the number of parcels; k = the number of participants) and fed to a principal component analysis. The first component was then considered and compared across the UP and MP by Pearson’s correlation. This approach was used to compare parcel-wise connectomics patterns expressed by rs-fMRI data built from different surfaces (UP and MP).

## Supplementary Information

Supplementary Information.

## Data Availability

The dataset analyzed in this study is stored at cnda.wustl.edu; Restrictions apply to the availability of the dataset which was analyzed in the current study under license from the cnda center in Washington University in St. Louis. The data-set in not publicly available, however it could be made available upon reasonable usage request from MC along with the permission of the cnda center (cnda.wustl.edu).

## Abbreviations

fMRI: Functional magnetic resonance imaging
T1w: T1-weighted
T2w: T2-weighted
BOLD: Blood oxygen level dependent
FC: Functional connectivity
UP: Unimodal pipeline
MP: Multimodal pipeline
