# Supplementary material for: Different MRI structural processing methods do not impact functional connectivity computation
Source: Sci Rep. 2023 May 26;13:8589. doi: 10.1038/s41598-023-34645-3 (PMC10219948; doi:10.1038/s41598-023-34645-3)

**Supplementary Table 1 Cortical Thickness.** Differences are reported for the different processing pipelines and hemispheres. Italics mark significant p-values (Bonferroni corrected).

| Regions | **Left hemisphere** | | |  | **Right hemisphere** | | |
| --- | --- | --- | --- | --- | --- | --- | --- |
|  | Mean ± SD (mm) | | *p*-value |  | Mean ± SD (mm) | | *p*-value |
|  | Unimodal | Multimodal |  |  | Unimodal | Multimodal |  |
| Banks of superior temporal sulcus | 2.57±0.14 | 2.63±0.16 | *<.0014* |  | 2.63±0.16 | 2.69±0.17 | *<.0014* |
| Caudal Anterior Cingulate cortex | 2.79±0.20 | 2.76±0.22 | .072 |  | 2.61±0.21 | 2.63±0.23 | .237 |
| Caudal Middle Frontal gyrus | 2.75±0.12 | 2.95±0.13 | *<.0014* |  | 2.7±0.12 | 2.94±0.14 | *<.0014* |
| Cuneus cortex | 1.88±0.12 | 2.03±0.15 | *<.0014* |  | 1.90±0.13 | 1.94±0.16 | *<.0014* |
| Entorhinal cortex | 3.41±0.26 | 3.57±0.26 | *<.0014* |  | 3.64±0.29 | 3.75±0.27 | *<.0014* |
| Fusiform gyrus | 2.79±0.12 | 2.97±0.14 | *<.0014* |  | 2.79±0.13 | 2.9±0.14 | *<.0014* |
| Inferior parietal lobule | 2.55±0.11 | 2.68±0.15 | *<.0014* |  | 2.59±0.11 | 2.67±0.15 | *<.0014* |
| Inferior temporal gyrus | 2.88±0.11 | 3.05±0.12 | *<.0014* |  | 2.87±0.13 | 3.01±0.14 | *<.0014* |
| Isthmus cingulate cortex | 2.42±0.16 | 2.51±0.16 | *<.0014* |  | 2.47±0.19 | 2.54±0.21 | *<.0014* |
| Lateral occipital cortex | 2.18±0.13 | 2.33±0.14 | *<.0014* |  | 2.25±0.12 | 2.37±0.14 | *<.0014* |
| Lateral orbitofrontal gyrus | 2.82±0.13 | 3.11±0.13 | *<.0014* |  | 2.80±0.14 | 3.10±0.15 | *<.0014* |
| Lingual gyrus | 2.01±0.11 | 2.15±0.13 | *<.0014* |  | 2.04±0.12 | 2.1±0.14 | *<.0014* |
| Orbitofrontal gyrus | 2.56±0.14 | 2.84±0.17 | *<.0014* |  | 2.55±0.16 | 2.85±0.18 | *<.0014* |
| Middle temporal gyrus | 2.97±0.12 | 3.04±0.14 | *<.0014* |  | 2.99±0.13 | 3.04±0.15 | *<.0014* |
| Parahippocampal gyrus | 2.85±0.24 | 3.09±0.26 | *<.0014* |  | 2.78±0.27 | 2.98±0.30 | *<.0014* |
| Paracentral lobule | 2.49±0.14 | 2.63±0.19 | *<.0014* |  | 2.54±0.14 | 2.66±0.18 | *<.0014* |
| Pars opercularis | 2.72±0.12 | 2.90±0.13 | *<.0014* |  | 2.73±0.16 | 2.95±0.17 | *<.0014* |
| Pars orbitalis | 2.83±0.18 | 3.11±0.18 | *<.0014* |  | 2.83±0.19 | 3.11±0.20 | *<.0014* |
| Pars triangularis | 2.59±0.15 | 2.76±0.16 | *<.0014* |  | 2.56±0.14 | 2.79±0.16 | *<.0014* |
| Pericalcarine cortex | 1.60±0.15 | 1.78±0.18 | *<.0014* |  | 1.60±0.14 | 1.73±0.16 | *<.0014* |
| Postcentral gyrus | 2.17±0.12 | 2.27±0.14 | *<.0014* |  | 2.16±0.14 | 2.25±0.17 | *<.0014* |
| Posterior cingulate cortex | 2.59±0.15 | 2.64±0.19 | *<.0014* |  | 2.56±0.13 | 2.65±0.17 | *<.0014* |
| Precentral gyrus | 2.73±0.11 | 2.89±0.14 | *<.0014* |  | 2.67±0.17 | 2.84±0.19 | *<.0014* |
| Precuneus | 2.47±0.11 | 2.58±0.14 | *<.0014* |  | 2.47±0.12 | 2.52±0.14 | *<.0014* |
| Rostral Anterior Cingulate Cortex | 3.02±0.18 | 3.25±0.15 | *<.0014* |  | 3.00±0.18 | 3.26±0.18 | *<.0014* |
| Rostral middle frontal gyrus | 2.5±0.12 | 2.66±0.13 | *<.0014* |  | 2.46±0.09 | 2.70±0.11 | *<.0014* |
| Superior frontal gyrus | 2.9±0.11 | 3.10±0.14 | *<.0014* |  | 2.88±0.12 | 3.13±0.14 | *<.0014* |
| Superior parietal lobule | 2.29±0.12 | 2.34±0.15 | *<.0014* |  | 2.28±0.12 | 2.29±0.16 | .802 |
| Superior temporal gyrus | 2.91±0.14 | 2.95±0.16 | .002 |  | 2.94±0.13 | 3.00±0.16 | *<.0014* |
| Supramargina gyrus | 2.70±0.12 | 2.77±0.13 | *<.0014* |  | 2.67±0.12 | 2.74±0.14 | *<.0014* |
| Frontal pole | 2.83±0.26 | 3.05±0.32 | *<.0014* |  | 2.82±0.24 | 3.08±0.25 | *<.0014* |
| Temporal pole | 3.72±0.24 | 3.99±0.26 | *<.0014* |  | 3.87±0.27 | 4.11±0.27 | *<.0014* |
| Transverse temporal gyrus | 2.51±0.20 | 2.61±0.19 | *<.0014* |  | 2.55±0.23 | 2.67±0.24 | *<.0014* |
| Insula | 3.13±0.15 | 3.37±0.15 | *<.0014* |  | 3.17±0.12 | 3.41±0.13 | *<.0014* |

**Supplementary Table 2 Cortical Volumes.** Differences are reported for the different processing pipelines and hemispheres. Italics mark significant p-values (Bonferroni corrected).

| Regions | **Left hemisphere** | | |  | **Right hemisphere** | | |
| --- | --- | --- | --- | --- | --- | --- | --- |
|  | Mean ± SD (mm) | | *p*-value |  | Mean ± SD (mm) | | *p*-value |
|  | Unimodal | Multimodal |  |  | Unimodal | Multimodal |  |
| Banks of superior temporal sulcus | 2590±445 | 2632±441 | *<.0014* |  | 2441±348 | 2476±345 | *<.0014* |
| Caudal Anterior Cingulate cortex | 1980±362 | 2032±415 | .003 |  | 2124±454 | 2251±530 | *<.0014* |
| Caudal Middle Frontal gyrus | 6683±860 | 7166±889 | *<.0014* |  | 6344±990 | 6927±1124 | *<.0014* |
| Cuneus cortex | 3077±551 | 3326 ±583 | *<.0014* |  | 3319±498 | 3295±522 | .261 |
| Entorhinal cortex | 2102±309 | 2209±329 | *<.0014* |  | 2124±314 | 2203±342 | *<.0014* |
| Fusiform gyrus | 10113±912 | 10915±982 | *<.0014* |  | 9737±1141 | 10270±1193 | *<.0014* |
| Inferior parietal lobule | 12790±1604 | 13543±1733 | *<.0014* |  | 15843±1752 | 16439±1865 | *<.0014* |
| Inferior temporal gyrus | 11739±1708 | 12628±1793 | *<.0014* |  | 11265±1338 | 12003±1419 | *<.0014* |
| Isthmus cingulate cortex | 2692±363 | 2820±370 | *<.0014* |  | 2548±338 | 2664±363 | *<.0014* |
| Lateral occipital cortex | 12378±1402 | 13387±1518 | *<.0014* |  | 12553±1340 | 13320±1410 | *<.0014* |
| Lateral orbitofrontal gyrus | 7854±759 | 8744±832 | *<.0014* |  | 7686±745 | 8544±821 | *<.0014* |
| Lingual gyrus | 6406±914 | 6864±1013 | *<.0014* |  | 7003±1069 | 7102±1136 | .011 |
| Orbitofrontal gyrus | 5314±580 | 5985±665 | *<.0014* |  | 5471±629 | 6225±713 | *<.0014* |
| Middle temporal gyrus | 12506±1624 | 13138±1745 | *<.0014* |  | 13166±1621 | 13771±1725 | *<.0014* |
| Parahippocampal gyrus | 2188±238 | 2385±260 | *<.0014* |  | 2075±294 | 2253±303 | *<.0014* |
| Paracentral lobule | 3524 ±443 | 3727±510 | *<.0014* |  | 3940±536 | 4155±588 | *<.0014* |
| Pars opercularis | 5149±931 | 5501±1004 | *<.0014* |  | 4299±560 | 4672±573 | *<.0014* |
| Pars orbitalis | 2577±380 | 2880±422 | *<.0014* |  | 3042±379 | 3386±423 | *<.0014* |
| Pars triangularis | 4045±647 | 4332±739 | *<.0014* |  | 4661±675 | 5090±738 | *<.0014* |
| Pericalcarine cortex | 2042±506 | 2236±559 | *<.0014* |  | 2247±463 | 2396±502 | *<.0014* |
| Postcentral gyrus | 9822±1238 | 10271±1340 | *<.0014* |  | 9360±1147 | 9711±1299 | *<.0014* |
| Posterior cingulate cortex | 3317±455 | 3384±510 | .002 |  | 3309±427 | 3454±455 | *<.0014* |
| Precentral gyrus | 14240±1490 | 15122±1669 | *<.0014* |  | 13695±1554 | 14626±1741 | *<.0014* |
| Precuneus | 9918±1046 | 10442±1130 | *<.0014* |  | 10339±1166 | 10604±1202 | *<.0014* |
| Rostral Anterior Cingulate Cortex | 2714±429 | 3043±515 | *<.0014* |  | 2016±417 | 2291±484 | *<.0014* |
| Rostral middle frontal gyrus | 16668±1989 | 17787±2304 | *<.0014* |  | 17526±2087 | 19276±2354 | *<.0014* |
| Superior frontal gyrus | 23827±2563 | 25680±3092 | *<.0014* |  | 23342±2537 | 25524±2960 | *<.0014* |
| Superior parietal lobule | 13432±1406 | 13674±1642 | .003 |  | 13377±1733 | 13253±1886 | .145 |
| Superior temporal gyrus | 13471±1737 | 13900±1870 | *<.0014* |  | 12454±1469 | 12914±1579 | *<.0014* |
| Supramargina gyrus | 11848±1437 | 12335±1483 | *<.0014* |  | 10617±1721 | 10990±1784 | *<.0014* |
| Frontal pole | 1062±204 | 1155±248 | *<.0014* |  | 1273±177 | 1408±195 | *<.0014* |
| Temporal pole | 2565±339 | 2839±375 | *<.0014* |  | 2695±352 | 2958±393 | *<.0014* |
| Transverse temporal gyrus | 1190±200 | 1252±211 | *<.0014* |  | 937±140 | 991±153 | *<.0014* |
| Insula | 7224±659 | 7785±711 | *<.0014* |  | 7207±741 | 7726±810 | *<.0014* |

**Supplementary Table 3 Gyrification Index.** Differences are reported for the different processing pipelines and hemispheres. Italics mark significant p-values (Bonferroni corrected).

| Regions | **Left hemisphere** | | |  | **Right hemisphere** | | |
| --- | --- | --- | --- | --- | --- | --- | --- |
|  | Mean ± SD (mm) | | *p*-value |  | Mean ± SD (mm) | | *p*-value |
|  | Unimodal | Multimodal |  |  | Unimodal | Multimodal |  |
| Banks of superior temporal sulcus | 3.61±0.21 | 3.55±0.20 | *<.0014* |  | 3.59±0.22 | 3.53±0.21 | .002 |
| Caudal Anterior Cingulate cortex | 1.87±0.07 | 1.86±0.08 | .020 |  | 1.94±0.09 | 1.92±0.08 | *<.0014* |
| Caudal Middle Frontal gyrus | 3.20±0.18 | 3.16±0.17 | .005 |  | 3.15±0.17 | 3.12±0.17 | .002 |
| Cuneus cortex | 2.95±0.17 | 2.88±0.17 | *<.0014* |  | 3.06±0.18 | 2.96±0.19 | *<.0014* |
| Entorhinal cortex | 2.51±0.12 | 2.43±0.10 | *<.0014* |  | 2.58±0.12 | 2.49±0.14 | *<.0014* |
| Fusiform gyrus | 2.66±0.09 | 2.60±0.09 | *<.0014* |  | 2.64±0.10 | 2.57±0.10 | *<.0014* |
| Inferior parietal lobule | 3.25±0.15 | 3.20±0.14 | *<.0014* |  | 3.24±0.12 | 3.19±0.11 | *<.0014* |
| Inferior temporal gyrus | 2.72±0.11 | 2.66±0.09 | *<.0014* |  | 2.64±0.10 | 2.59±0.10 | *<.0014* |
| Isthmus cingulate cortex | 2.71±0.16 | 2.67±0.14 | *<.0014* |  | 2.82±0.19 | 2.73±0.17 | *<.0014* |
| Lateral occipital cortex | 2.62±0.12 | 2.58±0.11 | *<.0014* |  | 2.62±0.11 | 2.55±0.11 | *<.0014* |
| Lateral orbitofrontal gyrus | 2.62±0.14 | 2.56±0.12 | *<.0014* |  | 2.54±0.13 | 2.47±0.13 | *<.0014* |
| Lingual gyrus | 2.79±0.11 | 2.72±0.11 | *<.0014* |  | 2.85±0.15 | 2.75±0.16 | *<.0014* |
| Orbitofrontal gyrus | 2.05±0.09 | 2.01±0.09 | *<.0014* |  | 2.07±0.10 | 2.02±0.10 | *<.0014* |
| Middle temporal gyrus | 3.37±0.20 | 3.29±0.19 | *<.0014* |  | 3.27±0.17 | 3.22±0.15 | .008 |
| Parahippocampal gyrus | 2.73±0.15 | 2.66±0.15 | *<.0014* |  | 2.79±0.13 | 2.72±0.14 | *<.0014* |
| Paracentral lobule | 2.32±0.09 | 2.29±0.10 | *<.0014* |  | 2.35±0.12 | 2.31±0.11 | *<.0014* |
| Pars opercularis | 4.24±0.32 | 4.17±0.25 | .008 |  | 4.28±0.31 | 4.21±0.30 | *<.0014* |
| Pars orbitalis | 2.98±0.20 | 2.93±0.17 | .009 |  | 2.95±0.21 | 2.88±0.21 | *<.0014* |
| Pars triangularis | 3.76±0.27 | 3.70±0.21 | *<.0014* |  | 3.73±0.29 | 3.66±0.24 | *<.0014* |
| Pericalcarine cortex | 2.83±0.14 | 2.75±0.14 | *<.0014* |  | 2.93±0.17 | 2.83±0.18 | *<.0014* |
| Postcentral gyrus | 3.52±0.14 | 3.48±0.13 | <.0014 |  | 3.49±0.15 | 3.44±0.16 | *<.0014* |
| Posterior cingulate cortex | 2.16±0.11 | 2.14±0.11 | .008 |  | 2.20±0.14 | 2.16±0.12 | *<.0014* |
| Precentral gyrus | 3.47±0.16 | 3.43±0.14 | .003 |  | 3.43±0.15 | 3.39±0.16 | *<.0014* |
| Precuneus | 2.89±0.15 | 2.85±0.13 | *<.0014* |  | 3.02±0.18 | 2.94±0.17 | *<.0014* |
| Rostral Anterior Cingulate Cortex | 2.01±0.09 | 1.98±0.08 | *<.0014* |  | 2.08±0.11 | 2.04±0.10 | *<.0014* |
| Rostral middle frontal gyrus | 2.76±0.14 | 2.72±0.13 | *<.0014* |  | 2.78±0.14 | 2.74±0.13 | *<.0014* |
| Superior frontal gyrus | 2.18±0.09 | 2.15±0.09 | *<.0014* |  | 2.23±0.09 | 2.21±0.09 | *<.0014* |
| Superior parietal lobule | 2.98±0.12 | 2.93±0.11 | *<.0014* |  | 2.98±0.14 | 2.92±0.14 | *<.0014* |
| Superior temporal gyrus | 4.11±0.25 | 4.04±0.23 | *<.0014* |  | 4.06±0.23 | 4.00±0.22 | *<.0014* |
| Supramargina gyrus | 3.60±0.15 | 3.55±0.15 | *<.0014* |  | 3.57±0.15 | 3.52±0.15 | *<.0014* |
| Frontal pole | 2.05±0.09 | 2.00±0.08 | *<.0014* |  | 2.09±0.09 | 2.03±0.09 | *<.0014* |
| Temporal pole | 2.39±0.14 | 2.31±0.11 | *<.0014* |  | 2.36±0.13 | 2.27±0.12 | *<.0014* |
| Transverse temporal gyrus | 4.70±0.29 | 4.64±0.27 | .006 |  | 4.70±0.31 | 4.64±0.32 | .002 |
| Insula | 4.34±0.28 | 4.26±0.23 | *<.0014* |  | 4.30±0.30 | 4.24±0.28 | *<.0014* |

**Supplementary Figure 1.** Vertex-level cortical thickness differences between unimodal and multimodal pipelines in a sub-cohort of participants (n = 44) acquired with the same MRI protocol. The z map of the cortical thickness difference between pipelines from this subgroup was highly correlated with the z map from the whole dataset (Pearson’s *r* = 0.83; *p* < 0.001).


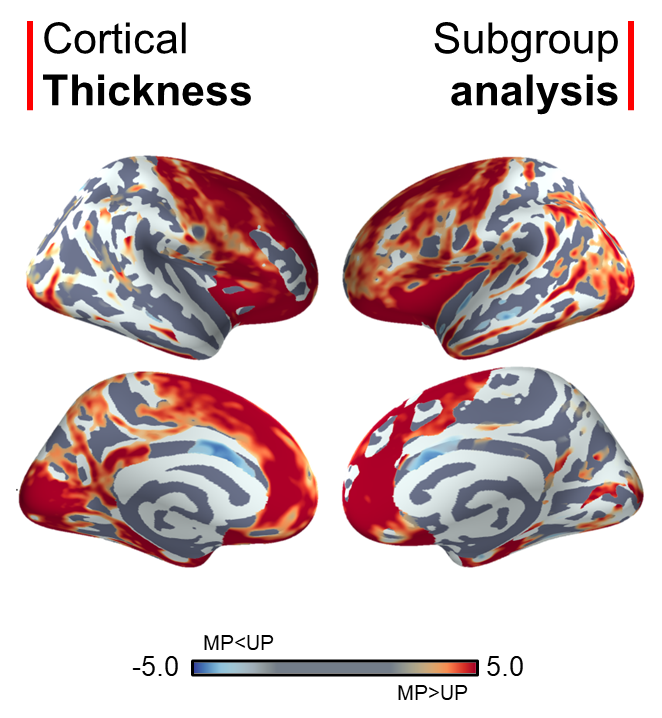

Supplement: Supplementary file 1 — Supplementary Information. [file 41598_2023_34645_MOESM1_ESM.docx]
